# Long-term prognostic value of stress perfusion cardiovascular magnetic resonance in patients without known coronary artery disease

**DOI:** 10.1186/s12968-021-00737-0

**Published:** 2021-04-08

**Authors:** Théo Pezel, Thierry Unterseeh, Marine Kinnel, Thomas Hovasse, Francesca Sanguineti, Solenn Toupin, Stéphane Champagne, Philippe Garot, Jérôme Garot

**Affiliations:** 1 CMR Department, Institut Cardiovasculaire Paris Sud, Cardiovascular Magnetic Resonance Laboratory, Hôpital Privé Jacques CARTIER, Ramsay Santé, 6 Avenue du Noyer Lambert, 91300 Massy, France; 2 Division of Cardiology, Johns Hopkins University, Baltimore, MD 21287-0409 USA; 3 Siemens Healthcare France, 93200 Saint-Denis, France

**Keywords:** Cardiovascular magnetic resonance, Stress testing, Ischemia, Unrecognized myocardial infarction, Perfusion

## Abstract

**Background:**

To assess the incremental long-term prognostic value of vasodilator stress perfusion cardiovascular magnetic resonance (CMR) in patients without known coronary artery disease (CAD).

**Methods:**

Between 2010 and 2011, consecutive patients with cardiovascular risk factors without known CAD referred for stress CMR were followed for the occurrence of major adverse cardiac events (MACE), defined by cardiovascular mortality or recurrent non-fatal myocardial infarction (MI). Uni- and multivariable Cox regressions were performed to determine the prognostic value of ischemia and unrecognized MI defined by sub-endocardial or transmural late gadolinium enhancement (LGE).

**Results:**

Among 2,295 patients without known CAD, 2058 (89.7%) (71.2 ± 12.5 years; 37.5% males) completed the follow-up (median [IQR]: 8.3 [7.3–8.7] years), and 203 had MACE (9.9%). Using Kaplan–Meier analysis, ischemia and unrecognized MI were associated with MACE (hazard ratio, HR: 4.64 95% CI: 3.69–6.17 and HR: 2.88; 95% CI: 2.08–3.99, respectively; both p < 0.001). In multivariable stepwise Cox regression, ischemia and unrecognized MI were independent predictors of MACE (HR = 3.71; 95% CI 2.73–5.05, *p* < 0.001 and HR = 1.73; 95% CI 1.22–2.45, *p* = 0.002; respectively) and cardiovascular mortality (HR: 3.13; 95% CI: 2.17–4.51, p < 0.001 and HR = 1.73; 95% CI 1.15–2.62, *p* = 0.009; respectively). The addition of ischemia and unrecognized MI led to an improved model discrimination for MACE (change in C statistic from 0.61 to 0.72; NRI = 0.431; IDI = 0.053).

**Conclusions:**

Inducible ischemia and unrecognized MI identified by stress CMR have incremental long term prognostic value for the incidence of MACE in patients without known CAD over traditional risk factors and left ventricular ejection fraction.

**Supplementary Information:**

The online version contains supplementary material available at 10.1186/s12968-021-00737-0.

## Introduction

The primary prevention to stratify cardiovascular risk of subjects without known cardiovascular disease (CAD) is crucial for public health and health care costs [1]. To date, decisions regarding the management of these individuals have relied mostly on the assessment of traditional risk factors. However, noninvasive cardiac stress testing may also play an important role for risk stratification, and therefore constitutes a cornerstone of the management of these subjects in the European and American guidelines [2, 3].

Stress cardiovascular magnetic resonance (CMR) imaging has emerged as a cost-effective modality for the diagnosis of CAD, and for risk stratification of cardiovascular events through the detection of both inducible myocardial ischemia and myocardial scar [4–7]. Prior studies have shown that beyond the major prognostic role of inducible ischemia, the depiction of an unrecognized myocardial infarction (MI) in individuals without known CAD is a strong predictor of cardiovascular events in the general population [8–11]. Recently, Nagel et al. demonstrated that a noninvasive diagnostic strategy based on stress CMR was noninferior to fractional flow reserve (FFR), in terms of outcomes in patients with suspected CAD [12].

Several large stress CMR prognostic studies have included subjects without known CAD [4, 6, 7, 11, 13, 14]. In addition, targeted prognostic data in those patients without known CAD were described in specific subpopulations such as asymptomatic, elderly or obese patients [5, 15–17]. However, the incremental prognostic value of the presence of inducible myocardial ischemia and unrecognized MI vs. traditional risk factors has not been evaluated.

We hypothesized that inducible myocardial ischemia and unrecognized MI assessed by stress CMR could identify patients at higher risk for cardiovascular event in primary prevention. This study aimed to assess the long-term prognostic value of vasodilator stress perfusion CMR in subjects without known CAD and to evaluate the incremental risk stratification of stress CMR over traditional risk factors and left ventricular ejection fraction (LVEF).

## Methods

### Study population

Between December 2010 and December 2011, we conducted a single-centre longitudinal study with retrospective enrollment of consecutive patients without known CAD, referred for vasodilator stress perfusion CMR. Exclusion criteria were: (i) history of CAD [percutaneous coronary intervention (PCI) or coronary artery bypass graft (CABG) or MI, defined by a history of MI on the medical records or presence of significant Q wave on 12-lead electrocardiogram (ECG) in a coronary territory]; (ii) contraindication to CMR (cerebral clips, metallic eye implant); (iii) contraindication to dipyridamole; (iv) known cardiomyopathy (e.g. hypertrophic, dilated, or infiltrative) and acute or chronic myocarditis; (v) known allergy to gadolinium-based contrast medium; and (vi) estimated glomerular filtration rate < 30 mL/min/1.73 m^2^. Clinical data were collected according to medical history and clinical examination on the day of stress CMR. All patients provided informed written consent. The study was approved by the local ethic committee of our institutions and conducted in accordance with the 1964 Declaration of Helsinki. This study followed the Strengthening the Reporting of Observational Studies in Epidemiology (STROBE) reporting guideline for cohort studies.

### Patients follow-up and clinical outcome

The follow-up consisted of a clinical visit as part of usual care (63%) or by direct contact with the subject or the referring cardiologist (37%). A clinical questionnaire with a detailed description of clinical study outcomes was filled out by three senior cardiologists. Data collection was ended on January 2020. The primary outcome was the occurrence of at least one of the combined major adverse cardiac events (MACE) defined as cardiovascular mortality or non-fatal MI. The secondary outcome was cardiovascular mortality. Clinical event adjudication was based on the follow-up clinical visit or contact, with a consensus reached by two senior cardiologists. Non-fatal MI was defined by typical angina of ≥ 20 min duration, ECG changes, and a rise in troponin or creatine kinase level above the 99 percentile of the upper reference limit [18]. Cardiovascular mortality was defined as sudden cardiac death with documented fatal arrhythmias or any death immediately preceded by acute MI, acute or exacerbation of heart failure, or stroke. All clinical events were defined according to the published standardized definitions [19]. In patients with multiple events, only the first event was considered for event-free survival analysis. According to guidelines, an hospitalization for heart failure was defined by symptoms and/or signs of heart failure with evidence of diastolic or systolic dysfunction by echocardiography and elevated levels of brain natriuretic peptide ((BNP) > 35 pg/ml and/or NT-proBNP > 125 pg/ml)) [20]. Ventricular tachycardia was defined as documented sustained ventricular tachycardia on 12-leads ECG. Elective late coronary revascularization was defined by a revascularization occurring > 90 days after CMR. For patients who underwent PCI < 90 days after the index examination, peri-procedural events (MI or cardiovascular mortality) [21] were not included in the analysis.

### CMR protocol

The detailed CMR protocol has already been published in our previous studies [15, 16]. CMR was performed on a 1.5 T CMR scanner (MAGNETOM Espree, Siemens Healthineers, Erlangen, Germany) with an 18-channel anterior body coil. Long-axis (2-, 3-, and 4-chamber) and short-axis cine images encompassing the left ventricle (LV) from base to apex were obtained with a segmented retrospectively gated balanced steady state free precession (bSSFP) sequence. Vasodilatation was induced with dipyridamole injected at 0.84 mg/kg over 3 min for all patients. At the end of dipyridamole infusion, a bolus of gadolinium-based contrast agent (0.1 mmol/kg, Dotarem®, Guerbet, France) was injected at a rate of 5.0 ml/s with an injector (Optistar® Elite, Mallinckrodt). Stress perfusion imaging was performed using an ECG-triggered saturation-prepared bSSFP sequence with the following typical parameters: repetition time/echo time (TR/TE) = 287/1.2 ms, acceleration factor = 2, field of view = 370 × 314 mm, matrix = 224 × 180, reconstructed pixel size = 1.7 × 1.7 × 8 mm. A series of six slices (four short-axis views: a 2-chamber and a 4-chamber view) were acquired every other heartbeat. Then, theophylline was injected intravenously (250 mg over 5 min) to null the effect of dipyridamole. Ten minutes after contrast injection, breath-hold contrast-enhanced 3D T1-weighted inversion-recovery gradient-echo sequence was acquired with the same prescriptions to detect late gadolinium enhancement (LGE). The inversion time was individually adjusted to null normal myocardium. In case of artifacts on LGE, additional 2D single-shot bSSFP images with phase sensitive inversion recovery reconstruction were acquired. Patients were asked to refrain from caffeine at least 12 h before CMR. Safety was studied with clinical monitoring 1 h after CMR to assess major adverse events. A 12-lead ECG was performed both before and after CMR examination.

### CMR image analysis

The *syngo*.via software (Siemens Healthineers) was used for image display and processing, and Hemolia (Clinigrid Inc., Paris, France) was used for reporting. LV volumes and function were quantified on the short-axis cine stack. Stress perfusion and LGE images were evaluated according to the 17-segment model of the American Heart Association [22]. The analysis of perfusion images was done visually by two experienced cardiologists (JG and FS) blinded to clinical and follow-up data. Inducible ischemia was defined as a subendocardial perfusion defect that (1) occurred in at least one myocardial segment, (2) persisted for at least three phases beyond peak contrast enhancement, (3) followed a coronary distribution, and (4) occurred in the absence of LGE in the same segment [13, 23–25]. An unrecognized MI was defined by LGE with ischemic patterns defined by subendocardial or transmural LGE [26]. The total number of ischemic segments was calculated for each patient. LGE was semi-quantitatively assessed using the number of LGE segments. Mild, moderate, and severe ischemia were defined as the involvement of 1–2, 3–5, and ≥ 6 myocardial segments, respectively.

### Statistical analysis

Continuous variables were expressed as mean ± standard deviation (SD) and categorical variables as frequency with percentage. Follow-up was presented as median and interquartile range (IQR). Differences between patients with and without inducible ischemia in terms of baseline clinical and CMR characteristics were compared using the Student’s t-test or the Wilcoxon rank-sum test for continuous variables and the chi-square or Fisher’s exact test for categorical variables, as appropriate. Normal distribution was assessed using the Shapiro–Wilk test. Cumulative incidence rates of individual and composite outcomes were estimated using the Kaplan–Meier method and compared with the log-rank test. The proportional hazard assumption was visually assessed using Schoenfeld residuals (Additional file 1: Figure S1). Data on patients who were lost to follow-up were censored at the time of the last contact. Cox proportional hazards methods were used to identify the predictors of MACE among patients with and without ischemia. The assumption of proportional hazards ratio (HR) was verified.

The different multivariable models used for adjustment were as follows:

Model 1: used a stepwise forward Cox regression strategy to select the strongest parsimonious set of clinical covariates for MACE and cardiovascular mortality, considering all clinical covariates with a p-value ≤ 0.2 on univariable screening (without the presence of ischemia and unrecognized MI).

Model 2: model 1 + presence of unrecognized MI.

Model 3: model 1 + presence of unrecognized MI and presence of ischemia.

The discriminative capacity of each model for predicting MACE was determined according to the Harrell’s C-statistic at baseline and after addition of CMR-assessed ischemia and MI. The additional predictive value of the presence of ischemia and MI was calculated by the Harrell’s C-statistic increment, the categorical net reclassification improvement (NRI), and the integrative discrimination index (IDI) [27]. NRI and IDI were computed at the end of follow-up using the R package “survIDINRI” [28].

In addition, the prognostic value of stress CMR in different subsamples of clinical interest was investigated by a Forest Plot. A two-tailed p-value < 0.05 was considered statistically significant. Statistical analysis was performed using R software, version 3.3.1 (R Project for Statistical Computing).

## Results

### Patients characteristics

**Fig. 1:**
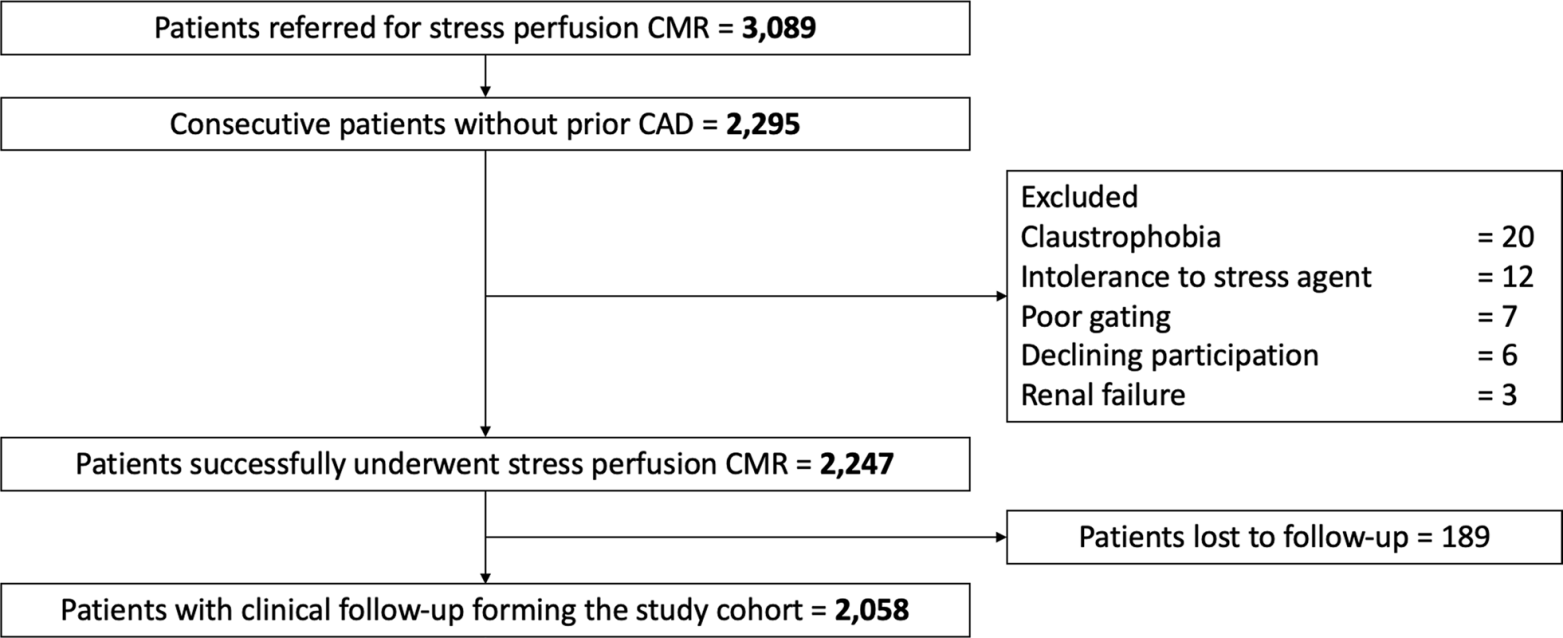
Flowchart. Flowchart of study patients

**Fig. 2:**
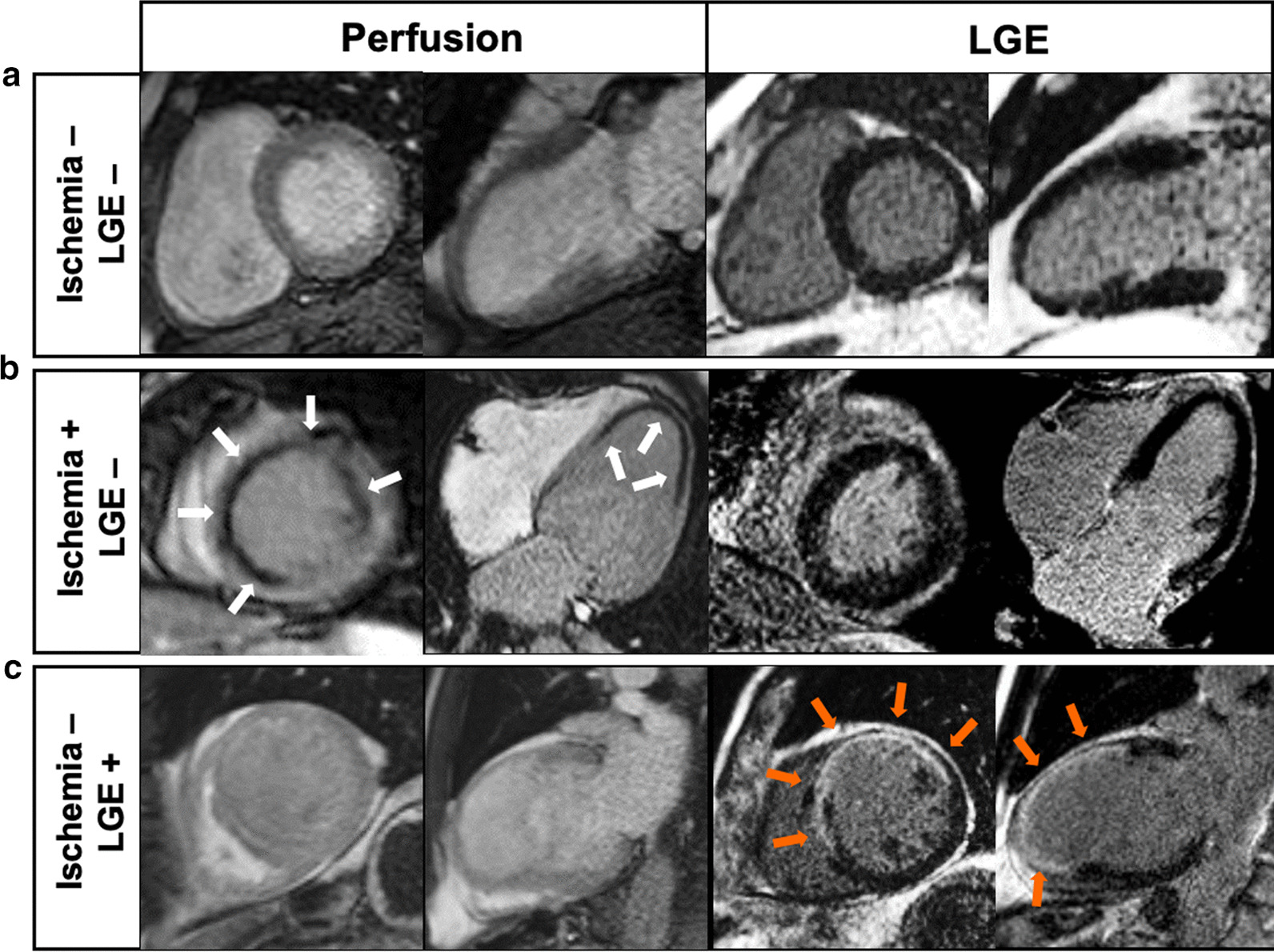
Examples of inducible ischemia on stress CMR in patients without prior CAD. **a** fifty-three-year-old female without prior CAD but with diabetes, dyslipidemia and hypertension. Stress CMR revealed no perfusion defect and no LGE, excluding the diagnosis of CAD. **b** Fifty-eight-year-old female without prior CAD but with diabetes and obesity referred for atypical chest pain. Stress CMR showed a subendocardial perfusion defect on the anteroseptal wall on first-pass perfusion images (white arrows) without myocardial scar on LGE, indicative of myocardial ischemia. Coronary angiography revealed a high-grade stenosis of the LAD. **c** Seventy-one-year-old male without prior CAD but with diabetes, hypertension and heredity referred for atypical chest pain. Stress CMR showed a transmural anteroseptal MI on *LGE (orange arrows)* without perfusion defect, therefore no ischemia. Coronary angiography confirmed the chronic occlusion of the LAD and the absence of other significant stenosis. *CAD *coronary artery disease, *LAD* left anterior descending, *LGE* late gadolinium enhancement, *RCA* right coronary artery

During the inclusion period, 2295 patients without known CAD were referred for dipyridamole stress CMR (Fig. 1). Amongst them, 2,058 patients completed the clinical follow-up and constituted our study cohort. Baseline subject characteristics and baseline CMR data are shown in Table 1. Among those 2,058 subjects (71.2 ± 12.5 years; 37.5% males), 66.0% had hypertension, 48.9% dyslipidemia, 35.3% obesity, 34.1% diabetes mellitus, 24.9% family history of CAD, and 21.2% were smokers. Most subjects were in sinus rhythm (99.6%). The overall study cohort had an LVEF of 52.6 ± 12.7%. An unrecognized MI was diagnosed by LGE with an ischemic pattern in 210 (10.2%) patients, and inducible ischemia was detected in 267 (13.0%) patients (Fig. 2).

Patients with ischemia were older (74.7 ± 11.4 vs. 70.8 ± 12.6 years, p < 0.001) and more commonly male (59.6% vs. 34.2%, p < 0.001). Patients with ischemia presented a higher cardiovascular risk using the ten-year risk for fatal CAD score [29] (3.4 [1.7–6.4]% vs. 2.4 [0.9–5.4]%, p < 0.001) and the Framingham Risk Score > 20% risk of CAD at 10 years [30] (56.9% vs. 31.9%, p < 0.001).

Of the 267 patients with ischemia, 196 (73.4%) underwent coronary angiography with early revascularization < 90 days after CMR. Among those, 9 patients were censored due to the recurrence of MI or cardiovascular mortality within 90 days after CMR.

### CMR study

Of 2295 patients without known CAD, 2247 (97.9%) completed the stress CMR protocol. Reasons for failure to complete CMR are presented in Fig. 1. No patient died during or shortly after CMR. There was one case of unstable angina and one patient with persistent atrial fibrillation, but no cases of transient ischemic attack, disabling stroke, ST elevation MI or sustained ventricular tachycardia after stress CMR. The main adverse events during or immediately after stress CMR were: headaches (N = 276, 12.3%), chest discomfort (N = 198, 8.8%), nausea or vomiting (N = 195, 8.7%), dizziness (N = 54, 2.4%) and angina with ECG evidence of ischemia (N = 42, 1.9%). For all patients, symptoms resolved quickly after an intravenous injection of theophylline and with additional sublingual nitrates and/or intravenous beta blockers in 29 patients (1.3%).

### Prognostic value

Among 2247 patients who underwent the stress CMR protocol, 2058 (91.6%) completed the follow-up with a median (IQR) follow-up of 8.3 (7.3–8.7) years. There were 203 MACE (9.9%), including 150 cardiovascular mortality (7.3%) and 53 non-fatal MI (2.6%). Furthermore, 296 all-cause mortality (14.4%), 116 hospitalizations for heart failure (5.6%), 105 elective late coronary revascularizations (5.1%) (5 CABG), and 41 sustained documented ventricular tachycardia (2.0%) were recorded. The annualized event rates for MACE and cardiovascular mortality, depending on the presence and severity of ischemia, are shown in Additional file 1: Figure S2.

**Fig. 3:**
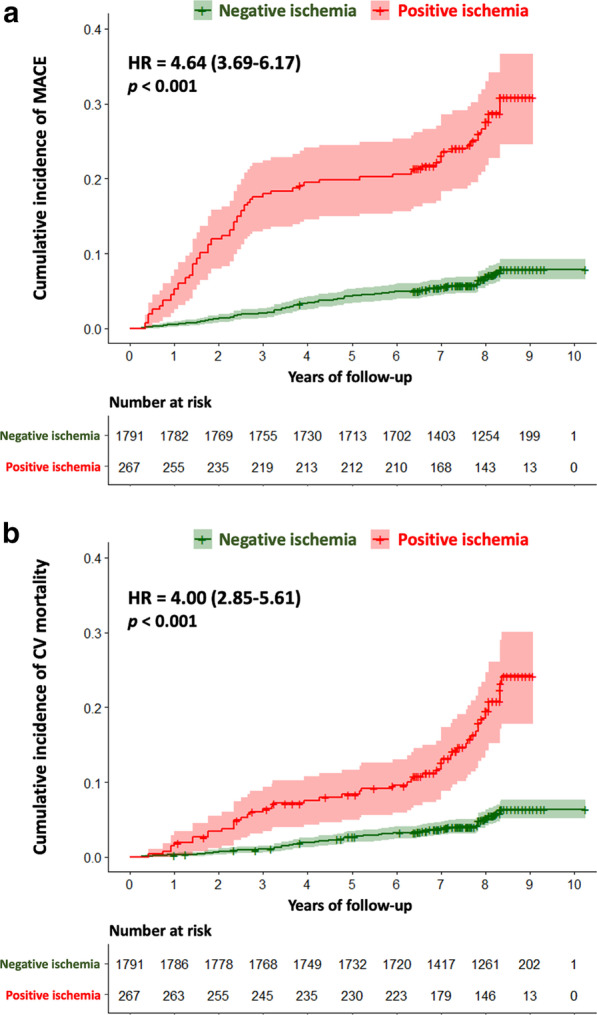
Kaplan–Meier curves for MACE (**a**) and cardiovascular mortality (**b**) stratified by the presence of ischemia. Kaplan Meier curves of MACE (cardiovascular mortality or non-fatal MI) as a function of length of follow-up for those with and without myocardial ischemia. Test comparing the two groups was based on the log-rank test

The univariable analysis of baseline patients and CMR characteristics for the prediction of MACE and cardiovascular mortality is shown in Table 2. Age, male gender, the presence of ischemia, the number of ischemic segments, the presence of unrecognized MI, LVEF and both LV end-diastolic and end-systolic volumes indexed were all significantly associated with MACE. Using Kaplan–Meier analysis, ischemia and unrecognized MI were associated with the occurrence of MACE (HR: 4.64 95% CI: 3.69–6.17 and HR: 2.88; 95% CI: 2.08–3.99, respectively; both p < 0.001) (Fig. 3). In addition, ischemia was associated with cardiovascular mortality (HR: 4.00; 95% CI: 2.85–5.61), non-fatal MI (HR: 6.20; 95% CI: 3.61–10.70) and all-cause mortality (HR: 2.74; 95% CI: 2.11–3.55, all p < 0.001; Additional file 1: Table S1). The presence of ischemia was significantly associated with MACE in men (HR: 5.29; 95% CI: 3.57–7.84) and in women (HR: 3.33; 95% CI: 2.08–5.34, both p < 0.001). The prognostic value of the presence of ischemia to predict MACE was significant in both asymptomatic and symptomatic patients (HR: 3.84; 95% CI: 2.53–5.82 and HR: 5.55; 95% CI: 3.74–8.24, respectively, both p < 0.001).

**Table 1:** Baseline and CMR characteristics of patients with and without inducible ischemia (N = 2058)

	All patients (N=2058)	No ischemia (N=1791)	Positive ischemia (N=267)	p value
Age, years	71.2 ± 12.5	70.8 ± 12.6	74.7 ± 11.4	**<0.001**
Males, n (%)	772 (37.5)	613 (34.2)	159 (59.6)	**<0.001**
Body mass index, kg/m²	29.4 ± 6.6	29.6 ± 6.7	28.3 ± 5.7	**<0.001**
Coronary artery disease risk factors, n (%)				
Diabetes mellitus	702 (34.1)	618 (34.5)	84 (31.5)	0.363
Hypertension	1358 (66.0)	1193 (66.6)	165 (61.8)	0.139
Dyslipidemia	1007 (48.9)	875 (48.9)	132 (49.4)	0.911
Current or previous smoking	436 (21.2)	378 (21.1)	58 (21.7)	0.881
Family history of coronary disease	512 (24.9)	450 (25.1)	62 (23.2)	0.551
Obesity^a^	726 (35.3)	644 (36.0)	82 (30.7)	0.109
Medical history of cardiovascular disease, n (%)				
Peripheral atheroma	52 (2.5)	45 (2.5)	7 (2.6)	0.662
Ischemic stroke	90 (4.4)	78 (4.4)	12 (4.5)	0.877
Pacemaker	10 (0.5)	9 (0.5)	1 (0.4)	1.000
Renal failure^b^	23 (1.1)	21 (1.2)	2 (0.7)	0.759
Prior hospitalization for heart failure	49 (2.4)	43 (2.4)	6 (2.3)	1.000
Indications to stress CMR (multiple possible), n (%)				
High cardiovascular disease risk^c^	724 (35.2)	572 (31.9)	152 (56.9)	**<0.001**
Symptomatic angina	530 (25.8)	447 (25.0)	83 (31.1)	**0.039**
Dyspnea	547 (26.6)	490 (27.4)	57 (21.3)	**0.046**
Inconclusive stress test	410 (19.9)	360 (20.1)	50 (18.7)	0.658
Inconclusive coronary CT angiogram^d^	26 (1.3)	24 (1.3)	2 (0.7)	0.566
Ten-year risk for fatal CAD (%)^e^	2.6 (1.1–5.6)	2.4 (0.9–5.4)	3.4 (1.7–6.4)	**<0.001**
Cardiac rhythm, n (%)				
Sinus rhythm	1507 (73.2)	1288 (71.9)	219 (82.0)	**<0.001**
Sinus rhythm with extrasystoles	542 (26.3)	495 (27.6)	47 (17.6)	
Atrial fibrillation/supraventricular arrhythmias	9 (0.4)	8 (0.4)	1 (0.4)	
LV ejection fraction, %	52.6 ± 12.7	52.9 ± 12.7	50.6 ± 12.7	**0.007**
LV end-diastolic volume index, ml/m^2^	81.1 ± 27.5	81.1 ± 27.3	81.5 ± 29.5	0.844
LV end-systolic volume index, ml/m^2^	40.6 ± 23.4	40.3 ± 23.3	42.3 ± 24.4	0.208
LV mass, g/m^2^	75.5 ± 7.7	72.9 ± 7.7	77.9 ± 7.8	**<0.001**
RV ejection fraction, %	65.9 ± 12.3	65.9 ± 12.3	65.8 ± 12.5	0.831
Presence of unrecognized MI, n (%)	210 (10.2)	135 (7.5)	75 (28.1)	**<0.001**
Number of segments of LGE	0.3 ± 1.0	0.2 ± 0.9	0.8 ± 1.6	**<0.001**
Number of segments of ischemia	0.3 ± 1.1	0.0 ± 0.0	2.6 ± 1.7	**<0.001**
Heart rate at baseline, beats/min	80 ± 13	80 ± 13	81 ± 15	0.688
Heart rate at stress, beats/min	92 ± 12	92 ± 11	94 ± 13	0.569
RPP at baseline, mmHg/beats/min	9.1 (7.6–10.8)	9.1 (7.6–10.7)	9.2 (7.6–10.9)	0.733
RPP at stress, mmHg/beats/min	10.4 (8.8–12.6)	10.4 (8.8–12.2)	11.2 (9.8–13.3)	0.363

Values are n (%), mean ± SD, or median (interquartile range)

*BMI* body mass index, *CAD* coronary artery disease, *CTA* computed tomography, *CMR* cardiovascular magnetic resonance, *LGE* late gadolinium enhancement, *LV* left ventricle, *MI* myocardial infarction, *RPP* rate-pressure product (pressure mmHg x Heart rate bpm)/1000, *RV* right ventricle, *SD* standard deviation

^a^Defined by body mass index ≥ 30 kg/m^2^

^b^Defined by estimated glomerular filtration rate < 60 ml/min/1.73 m^2^

^c^Defined by Framingham Risk Score > 20% of risk of CAD at 10 years

^d^Defined by coronary stenosis of unknown significance on coronary CT angiography

**Fig. 4:**
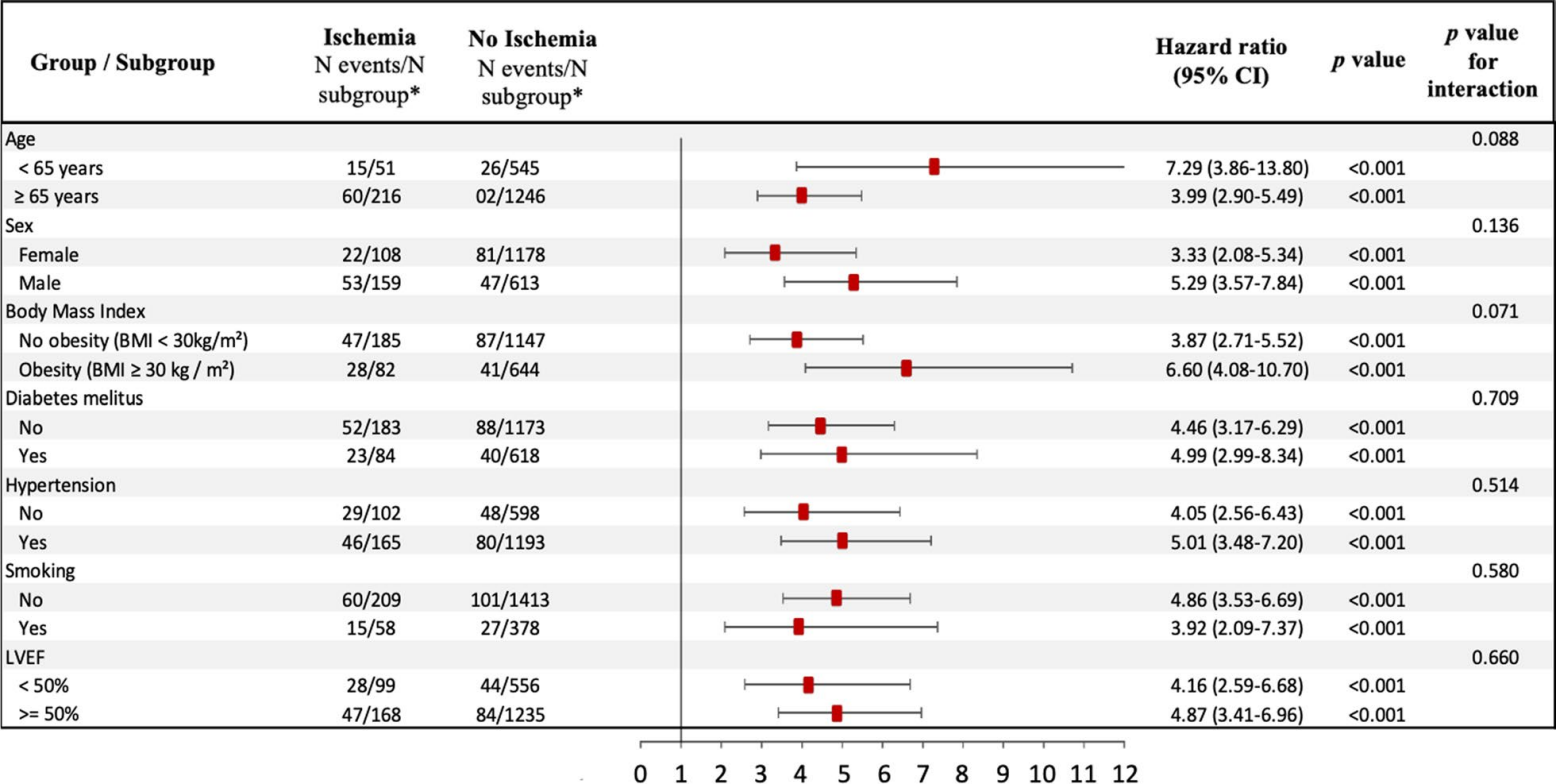
Subgroup analysis. Forest-plot of incidence of MACE based on the presence of ischemia in prespecified subgroups. ^*^N events/N subgroup: number of patients who had a major adverse clinical event (MACE) / number of patients in the subgroup

The prognostic value of ischemia remained consistent in all other subsamples of clinical interest such as diabetics and non-diabetics, and regardless of LVEF (Fig. 4). In addition, the presence of ischemia had a similar prognostic value regardless of the age (Additional file 1: Figure S3).

In multivariable stepwise Cox regression (model 2), the presence of ischemia and unrecognized MI were independent predictors of MACE (HR = 3.71; 95% CI 2.73–5.05, *p* < 0.001 and HR = 1.73; 95% CI 1.22–2.45, *p* = 0.002; respectively) and cardiovascular mortality (HR: 3.13; 95% CI: 2.17–4.51, p < 0.001 and HR = 1.73; 95% CI 1.15–2.62, *p* = 0.009; respectively) (Table 3).

**Table 2:** Univariable analysis of clinical and CMR characteristics for prediction of adverse events

	MACE		Cardiovascular mortality	
	Hazard ratio (95% CI)	p value	Hazard ratio (95% CI)	p value
Age	1.02 (1.01–1.03)	**0.002**	1.03 (1.02–1.05)	**< 0.001**
Male	1.68 (1.27–2.21)	** < 0.001**	1.60 (1.16–2.20)	**0.004**
Body mass index	0.98 (0.95–1.02)	0.311	1.01 (0.95–1.03)	0.485
Hypertension	0.83 (0.62–1.10)	0.190	0.88 (0.63–1.22)	0.431
Diabetes mellitus	0.86 (0.64–1.15)	0.307	0.87 (0.62–1.23)	0.429
Dyslipidemia	1.00 (0.76–1.32)	0.978	1.00 (0.73–1.38)	0.978
Current or previous smoking	0.96 (0.68–1.35)	0.811	0.81 (0.53–1.22)	0.309
Family history of coronary artery disease	0.72 (0.51–1.02)	0.064	0.61 (0.40–0.94)	**0.023**
Stroke	0.77 (0.37–1.83)	0.371	0.74 (0.30–1.19)	0.466
Renal failure	1.36 (0.44–4.26)	0.595	1.27 (0.31–5.12)	0.738
Peripheral atheroma	1.12 (0.29–3.37)	0.566	1.54 (0.34–4.45)	0.319
Prior hospitalization for heart failure	1.57 (0.74–3.34)	0.240	1.87 (0.83–4.24)	0.133
Presence of ischemia	4.64 (3.49–6.17)	** < 0.001**	4.00 (2.85–5.61)	**< 0.001**
Number of segments of ischemia	1.60 (1.51–1.70)	** < 0.001**	1.51 (1.40–1.62)	**< 0.001**
Presence of unrecognized MI	2.88 (2.08–3.99)	** < 0.001**	2.77 (1.89–4.06)	**< 0.001**
Number of segments of LGE	1.52 (1.41–1.63)	** < 0.001**	1.49 (1.37–1.62)	**< 0.001**
LV ejection fraction, per 10%	0.88 (0.79–0.97)	**0.014**	0.93 (0.82–1.05)	0.245
LV end-diastolic volume index, per 10 ml/m^2^	1.06 (1.01–1.11)	**0.025**	1.03 (0.97–1.09)	0.348
LV end-systolic volume index, per 10 ml/m^2^	1.06 (1.02–1.13)	**0.009**	1.04 (0.97–1.11)	0.252
RV ejection fraction, %	0.96 (0.79–1.19)	0.411	1.06 (0.78–1.53)	0.681

*CI* confidence interval, *CMR* cardiovascular magnetic resonance, *LGE* late gadolinium enhancement, *LV* left ventricle, *MACE* major adverse cardiac events, *MI* myocardial infarction, *RV* right ventricle

### Incremental prognostic value of stress CMR

For the prediction of MACE, we observed baseline C statistic values of 0.61 (95% CI, 0.56–0.65) for model 1 with stepwise variable selection. The addition of unrecognized MI significantly improved the C statistic to 0.66 (95% CI, 0.60–0.71; C statistic improvement for model 1: 0.05; NRI = 0. 252; IDI = 0.037). Furthermore, the addition of unrecognized MI and ischemia significantly improved the C statistic to 0.72 (95% CI, 0.69–0.76; C statistic improvement for model 1: 0.11; NRI = 0.431; IDI = 0.053) (Table 4).

**Table 3:** Multivariable cox regression analysis for the prediction of adverse events

	MACE		Cardiovascular mortality	
	Hazard ratio (95% CI)	p value	Hazard ratio (95% CI)	p value
*Model 1*^*a*^				
Age	1.03 (1.01–1.05)	**0.002**	1.04 (1.02–1.07)	** < 0.001**
Male	1.91 (1.32–2.76)	** < 0.001**	1.85 (1.22–2.82)	**0.004**
Family history of coronary artery disease	0.74 (0.49–1.10)	0.131	0.66 (0.41–1.05)	0.076
LV end-systolic volume index, per 10 ml/m^2^	2.70 (0.99–7.37)	0.053	3.81 (1.38–10.5)	**0.010**
*Model 2*^*b*^				
Presence of unrecognized MI	1.82 (1.28–2.49)	** < 0.001**	1.76 (1.18–2.66)	**0.007**
*Model 3*^*c*^				
Presence of unrecognized MI	1.73 (1.22–2.45)	**0.002**	1.73 (1.15–2.62)	**0.009**
Presence of ischemia	3.71 (2.73–5.05)	** < 0.001**	3.13 (2.17–4.51)	** < 0.001**

*CI* confidence interval, *MACE* major adverse cardiac events, *LV* left ventricle, *MI* myocardial infarction

^a^Covariates in the **model 1** by stepwise variable selection with entry and exit criteria set at the p ≤ 0.2 level: for MACE: age, male, hypertension, family history of CAD, LVEF per 10%, LV end-systolic volume index, per 10 ml/m^2^. for CV mortality: age, male, family history of CAD, family history of CAD

^b^Covariates in the **model 2**: model 1 with unrecognized MI

^c^Covariates in the **model 3**: model 1 with unrecognized MI and ischemia

**Table 4:** Discrimination and reclassification associated with ischemia and LGE for prediction of MACE

	MACE		
	C-index (95%CI)	NRI (95%CI)	IDI (95%CI)
Model 1 (stepwise selection)^a^	0.61 (0.56–0.65)	Reference	Reference
Model 2 (model 1 + unrecognized MI)^b^	0.66 (0.60–0.71)	0.252 (0.065–0.439)	0.037 (0.016–0.058)
Model 3 (model 1 + unrecognized MI and ischemia)^c^	0.72 (0.69–0.76)	0.431 (0.212–0.650)	0.053 (0.030–0.076)

*CI* confidence interval, *IDI* integrative discrimination index, *MACE* major adverse cardiac events, *MI* myocardial infarction, *NRI* net reclassification improvement

^a^Covariates in the **model 1** by stepwise variable selection with entry and exit criteria set at the p ≤ 0.2 level: age, male, hypertension, family history of coronary artery disease, LVEF per 10%, LV end-systolic volume index, per 10 ml/m^2^

^b^Covariates in the **model 2**: model 1 with unrecognized MI

^c^Covariates in the **model 3**: model 1 with unrecognized MI and ischemia

## Discussion

In this large single center study of patients without known CAD referred for stress CMR, our main findings demonstrate that: (1) inducible ischemia and unrecognized MI were independent long-term predictors of MACE and cardiovascular mortality; (2) the presence of inducible ischemia and unrecognized MI improved model discrimination for the prediction of MACE, after adjusting for covariates; (3) 13.0% of patients had inducible ischemia and 10.2% had unrecognized MI.

Previous studies in patients with suspected or known CAD reported that the prevalence of CMR-inducible ischemia ranged between 7 and 26% [4, 6, 15–17]. In the current study including low-risk patients without known CAD, the prevalence of inducible ischemia was 13.0%. The prevalence of unrecognized MI detected by CMR has been shown to range between 0.2 and 30% in the general population [9, 31, 32], about 15% in symptomatic patients [11], and less than 6% in asymptomatic patients with suspected CAD [17]. In the present study, the prevalence of unrecognized MI was 10.2%. Consistently with prior cohorts of patients without known CAD [4, 13, 15–17], the rate of MACE was 9.9% over a median follow-up of 8.3 years.

Stress-CMR inducible ischemia and unrecognized MI were independently associated with MACE in the sole subset of patients without known CAD. Such findings extend the aggregate data on the prognostic value of stress CMR [4, 6, 11, 15, 16, 33, 34]. Consistently, the extent of ischemia was a strong prognosticator of MACE and cardiovascular mortality, as already described in patients with known or suspected CAD [13]. Several studies have shown similar accuracy to diagnose CAD and predict cardiovascular events in men and women [35, 36]. In line with these data, the current study suggests a similar prognostic value of stress CMR in women and men.

Although some studies have suggested the prognostic value of stress CMR in patients without known CAD [5, 15–17], they have not evaluated the incremental prognostic value of stress CMR to predict cardiovascular events over traditional risk factors in this population. The current study demonstrates an incremental prognostic value of unrecognized MI to predict MACE above traditional cardiovascular risk factors and LVEF. This finding is in line with the recent SPINS registry of the Society for Cardiovascular Magnetic Resonance showing that presence of unrecognized MI portended a significant risk for cardiovascular events, independently of the presence of ischemia [11]. In the current, the addition of inducible ischemia to the model containing traditional risk factors and unrecognized MI, further improved the prognostic value for predicting MACE. These data highlight the importance of integrating both inducible ischemia and unrecognized MI in risk stratification models.

This addition of unrecognized MI and ischemia by stress CMR in risk stratification models led to an incremental prognostic value, as illustrated by a rise in the C-index from 0.61 to 0.66 and 0.72, respectively. Whereas this prognostic incremental value of stress CMR could be cost-effective remains to be evaluated. The SPINS registry [4] demonstrated that the average cost of ischemic testing was lower for stress CMR than other stress testing [37]. If cost-effective, the current data support the use of stress CMR to identify high-risk patients who could benefit from improved clinical and therapeutic management [38, 39].

### Study limitations

Our study has several limitations. First, the study was retrospective, with a risk of referral bias. Overall, 189 (8.4%) patients were lost to follow-up, which can be explained by a relatively long follow-up and the design of the study. However, the French National Registry of Death was carefully reviewed, which strengthens the data on mortality. Despite the good prognostic value of stress CMR in AF patients [40], the low proportion of AF patients referred for stress CMR is likely due to the reluctance of referring cardiologists. This study was not designed to compare the prognostic value in women and men. The analysis of CMR stress perfusion images was visual, which represents the most widely accepted clinical method with optimal diagnostic accuracy. Stress perfusion CMR protocol included six slices (4 short-axis views, and long-axis views) acquired every other heartbeat to optimize anatomical coverage of the LV, at the cost of a slight decrease in temporal resolution. This retrospective study could not capture all of the confounding factors regarding the association between management decisions after the stress CMR exam and patient risk. Finally, the extent of myocardial scar was assessed semi-quantitatively by the number of infarcted segments and not quantitatively by semi-automated methods.

## Conclusions

Stress perfusion CMR has a good discriminative long-term prognostic value in patients without known CAD. Stress-CMR inducible ischemia and unrecognized MI are independently associated with non-fatal MI and cardiovascular mortality over a long-term follow-up and offer incremental prognostic value over traditional risk factors.

## Supplementary Information

**Additional file 1: Figure S1.** The assessment of the proportional hazard assumption. **Figure S2.** Annualized event rates of MACE (A) and cardiovascular mortality (B) stratified by the extent of ischemia (N = 2058). **Table S1.** Univariable analysis of inducible myocardial ischemia for prediction of adverse events (N = 2058). **Figure S3.** Annualized event rates of MACE stratified by age and presence/absence of ischemia.

## Data Availability

All data generated or analysed during this study are included in this published article [and its Additional information files].

## Abbreviations

BMI: Body mass index
BNP: Brain natriuretic peptide
bSSFP: Balanced steady state free precession
CABG: Coronary artery bypass graft
CAD: Coronary artery disease
CI: Confidence interval
CMR: Cardiovascular magnetic resonance
ECG: Electrocardiogram
FFR: Fractional flow reserve
HR: Hazard ratio
IDI: Integrative discrimination index
LGE: Late gadolinium enhancement
LV: Left ventricle/left ventricular
LVEF: Left ventricular ejection fraction
MACE: Major adverse cardiac event
MI: Myocardial infarction
NRI: Net reclassification improvement
PCI: Percutaneous coronary intervention
SD: Standard deviation
IQR: Interquartile range
