# Supplementary material for: Long-term prognostic value of stress perfusion cardiovascular magnetic resonance in patients without known coronary artery disease
Source: J Cardiovasc Magn Reson. 2021 Apr 8;23:43. doi: 10.1186/s12968-021-00737-0 (PMC8028337; doi:10.1186/s12968-021-00737-0)
Supplement: Supplementary file 1 — Additional file 1: Figure S1. The assessment of the proportional hazard assumption. Figure S2. Annualized event rates of MACE (A) and cardiovascular mortality (B) stratified by the extent of ischemia (N = 2058). Table S1. Univariable analysis of inducible myocardial ischemia for prediction of adverse events (N = 2058). Figure S3. Annualized event rates of MACE stratified by age and presence/absence of ischemia. [file 12968_2021_737_MOESM1_ESM.docx]

**Additional Material**

**Table of Contents:**

- Additional file 1: Figure S1. The assessment of the proportional hazard assumption.
- Additional file 1: Figure S2. Annualized event rates of MACE (A) and cardiovascular mortality (B) stratified by the extent of ischemia (N=2,058).
- Additional file 1: Table S1. Univariable analysis of inducible myocardial ischemia for prediction of adverse events (N=2,058).
- Additional file 1: Figure S3. Annualized event rates of MACE stratified by age and presence/absence of ischemia.

Additional file 1: Figure S1

**The assessment of the proportional hazard assumption.**

The proportional hazard assumption was visually tested using Schoenfeld residuals for MACE. The proportional risk assumption is retained if the red line is always included between the dotted lines.

**
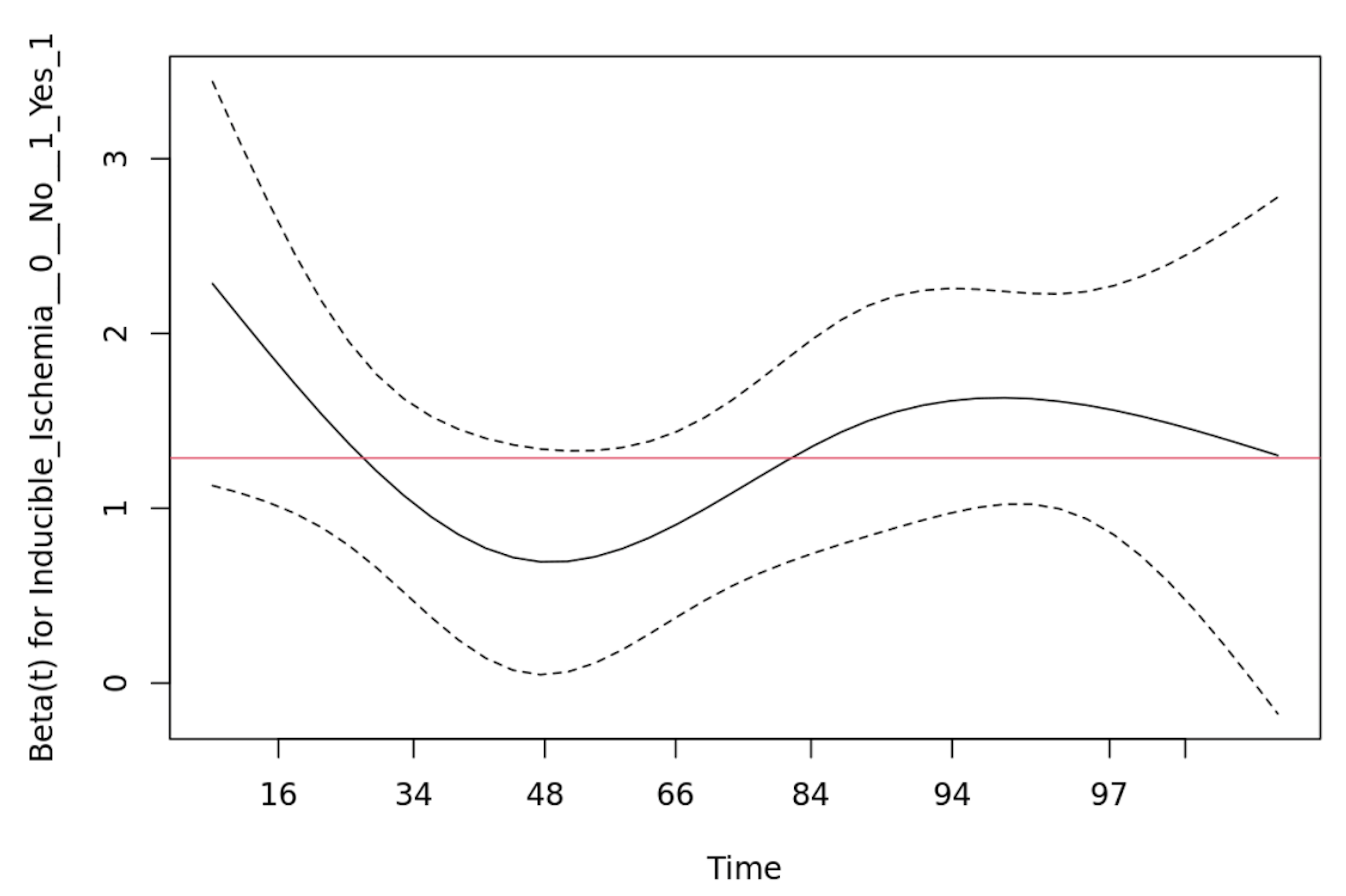
**

Additional file 1: Figure S2

**Annualized event rates of MACE (A) and cardiovascular mortality (B) stratified by the extent of ischemia (N=2,058).**

Mild, moderate, and severe ischemia was defined as the involvement of 1–2, 3–5, and ≥ 6 myocardial segments, respectively. Comparison tests were based on the Cochran-Armitage test for trend.

**
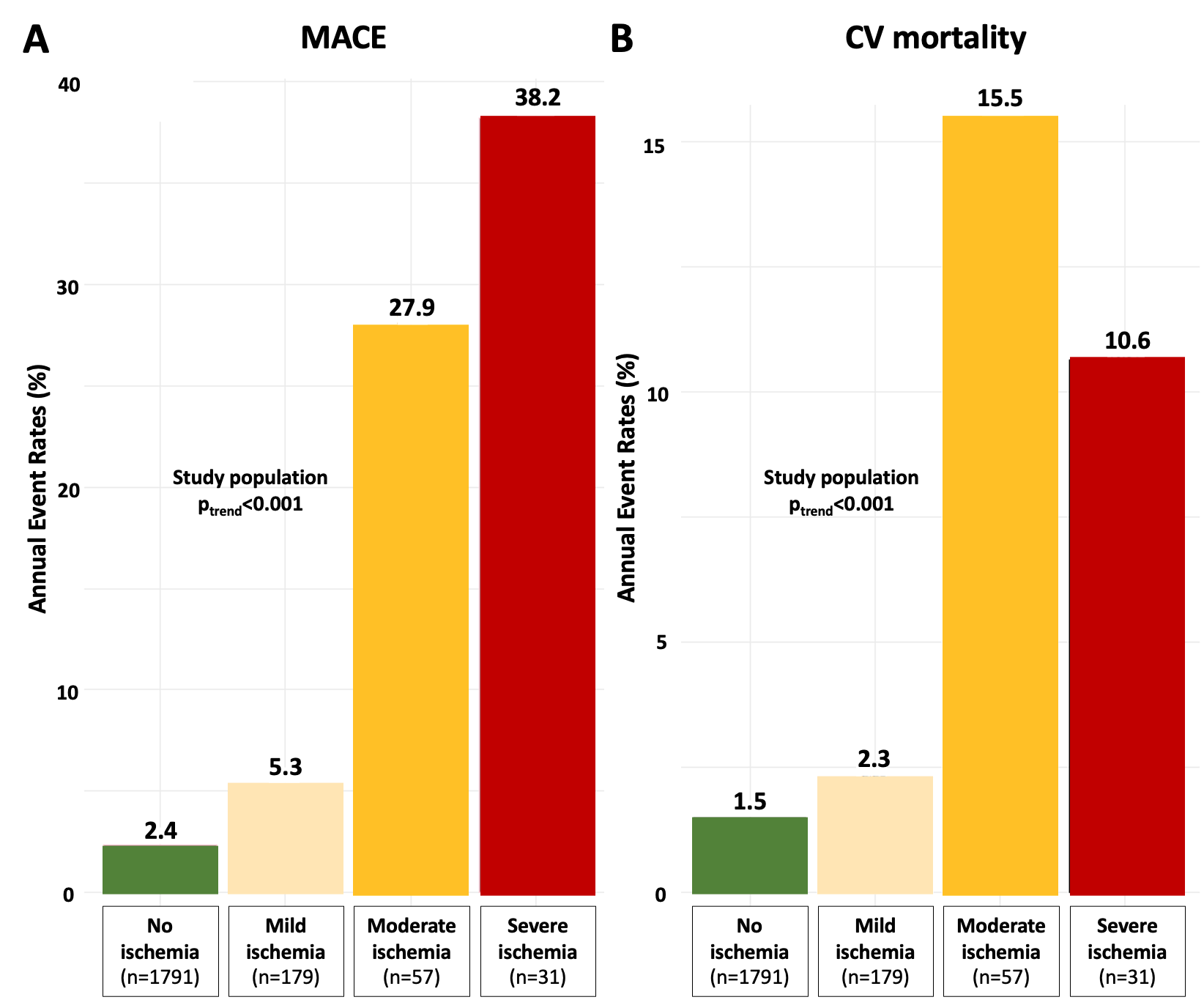
**

Additional file 1: Table S1

**Univariable analysis of inducible myocardial ischemia for prediction of adverse events (N=2,058).**

|  | **Univariable analysis** | |  |
| --- | --- | --- | --- |
|  | **Hazard Ratio (95% CI)** | **p value** |  |
|  |  |  |  |
| ***Primary outcome (MACE)*** |  |  |  |
| Cardiovascular mortality | 4.00 (2.85-5.61) | **<0.001** |  |
| Non-fatal MI | 6.20 (3.61-10.70) | **<0.001** |  |
|  |  |  |  |
| ***Secondary outcomes*** |  |  |  |
| All-cause mortality | 2.74 (2.11-3.55) | **<0.001** |  |
| Elective late coronary revascularization | 0.85 (0.45-1.58) | 0.604 |  |
| Hospitalization for heart failure | 1.43 (0.87-2.37) | 0.16 |  |
| Ventricular tachycardia | 1.69 (0.74-3.85) | 0.208 |  |
|  |  |  |  |

*Abbreviations: CI: confidence interval; MACE: major adverse cardiac events; MI: myocardial infarction.*

Additional file 1: Figure S3

**Annualized event rates of MACE stratified by age and presence/absence of ischemia.**

Annualized event rates of MACE are stratified by presence/absence of ischemia in different age categories: <50 years; 50-60 years; 60-70 years and > 70 years.

**
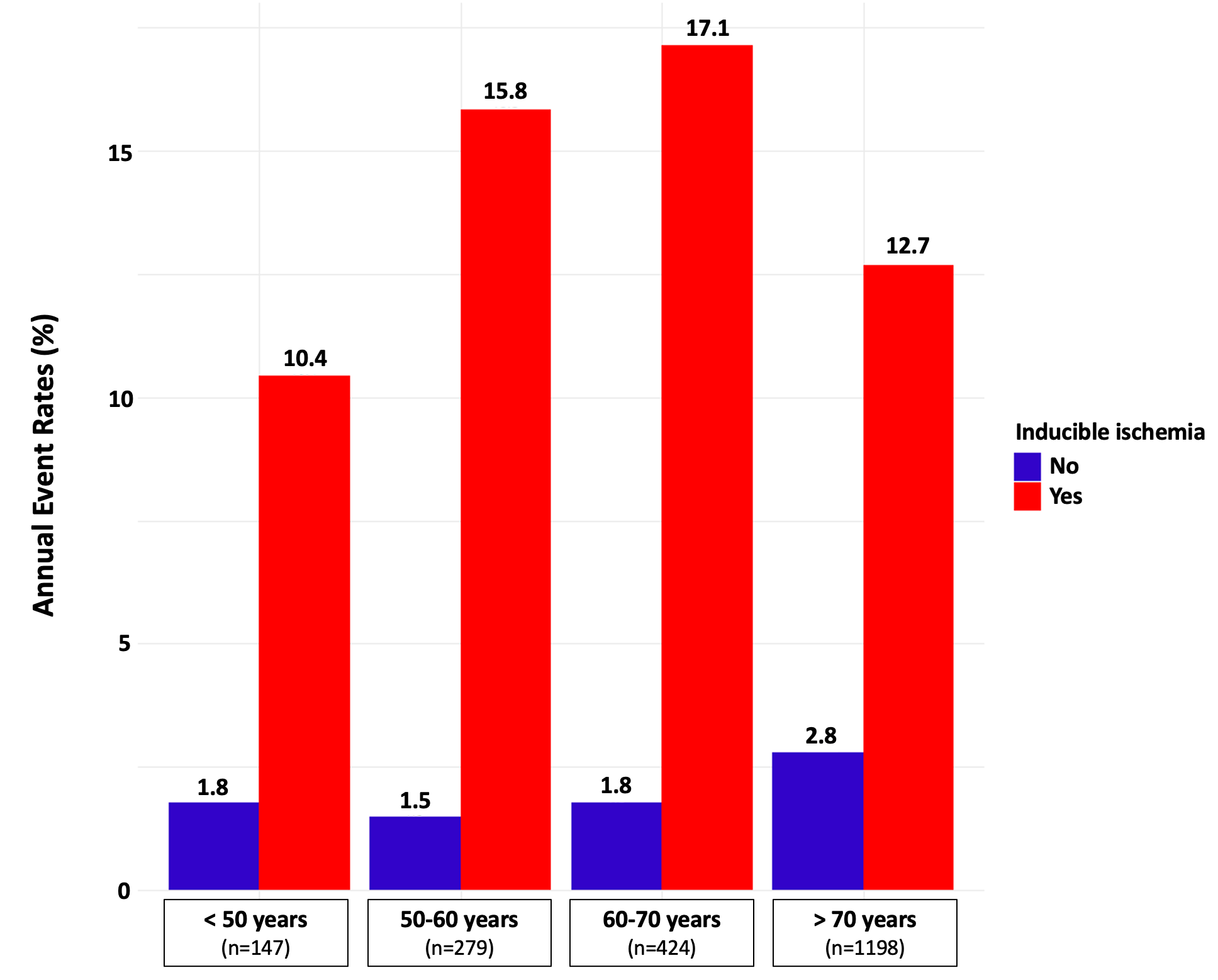
**
